# Nomogram for Stability Stratification of Small Intracranial Aneurysm Based on Clinical and Morphological Risk Factors

**DOI:** 10.3389/fneur.2020.598740

**Published:** 2021-01-15

**Authors:** Wei Zhu, Wenqiang Li, Zhongbin Tian, Mingqi Zhang, Yisen Zhang, Kun Wang, Ying Zhang, Xinjian Yang, Jian Liu

**Affiliations:** Department of Interventional Neuroradiology, Beijing Neurosurgical Institute and Beijing Tiantan Hospital, Capital Medical University, Beijing, China

**Keywords:** nomogram, intracranial aneurysm, instability stratification, unstable aneurysm, small aneurysm

## Abstract

**Background and Purpose:** Stability stratification of intracranial aneurysms (IAs) is crucial for individualized clinical management, especially for small IAs. We aim to develop and validate a nomogram based on clinical and morphological risk factors for individualized instability stratification of small IAs.

**Methods:** Six hundred fifty-eight patients with unstable (*n* = 293) and stable (*n* = 416) IAs <7 mm were randomly divided into derivation and validation cohorts. Twelve clinical risk factors and 18 aneurysm morphological risk factors were extracted. Combined with important risk factors, a clinical-morphological predictive nomogram was developed. The nomogram performance was evaluated in the derivation and the validation cohorts in terms of discrimination, calibration, and clinical usefulness.

**Results:** Five independent instability-related risk factors were included in the nomogram: location, irregularity, side/bifurcation type, flow angle, and height-to-width ratio. In the derivation cohort, the area under the curve (95% CI) of the nomogram was 0.803 (95% CI, 0.764–0.842), and good agreement between predicted instability risk and actual instability status could be detected in the calibration plot. The nomogram also exhibited good discriminations and calibration in the validation cohort: the area under the curve (95% CI) was 0.744 (95% CI, 0.677–0.812). Small IAs with scores <90 were considered to have low risk of instability, and those with scores of 90 or greater were considered to have high risk of instability.

**Conclusions:** The nomogram based on clinical and morphological risk factors can be used as a convenient tool to facilitate individualized decision-making in the management of small IAs.

## Introduction

Intracranial aneurysms (IAs) are common, with an estimated prevalence of 3.2 to 7% in the adult population (1, 2). However, the incidence of subarachnoid hemorrhage (SAH) caused by IAs rupture is <2%, which means that only a small portion of IAs will rupture (3). Nevertheless, acute IAs rupture is often associated with 30 to 67% mortality and 15 to 30% morbidity (4–6). Accordingly, the accurate identification of IAs stability is crucial to inform the management of patients with unruptured IAs. Size has been the most widely used surrogate for assessing IAs stability. The International Study of Unruptured Intracranial Aneurysms (ISUIA) and Unruptured Cerebral Aneurysm Study (UCAS) reported that the risk of IA rupture increases with its size, which led to the suggestion that small IAs (i.e., those <7 mm in diameter) are least likely to rupture than larger IAs (7, 8). However, in clinical practice, the percentage of ruptured small IAs in general is not low: according to numerous clinical reports, small IAs account for 35 to 50% of all ruptured IAs (9, 10). In addition, growing IAs are at high risk of rupture compared with those of stable size (11). As the number of incidental unruptured IAs increases, more treatment decisions are required. Thus, the stratification of IAs stability is meaningful, especially for small IAs.

Apart from size, many patient clinical characteristics (e.g., patient's age and hypertension) and IA morphology parameters [e.g., aspect ratio (AR) and size ratio (SR)] have been reported to be related to IA stability (12, 13). Because these pre-treatment clinical and morphological risk factors are easily accessible before administering treatment, they are ideal candidates to develop a predictive tool for IA instability assessment. Such a tool can provide valuable information that can help in the clinical decision-making process to determine the optimal treatment strategy, which may vary from conservative management to timely operation.

In this study, we aim to develop and validate a nomogram that incorporates both the clinical and morphological risk factors to enable personalized prediction of the stability of small IAs. Of all the available models, nomograms are easy to use and can provide an individualized, evidence-based, and highly accurate risk estimation. To our knowledge, this is the first attempt to develop a nomogram for instability stratification in small IAs.

## Materials and Methods

### Patient Population

This study was approved by the Institutional Review Board of Beijing Tiantan Hospital, and written informed consent was waived because this is a retrospective study. All participants were recruited from the Beijing Tiantan Hospital of Capital Medical University, between June 2014 and June 2018. The patients' information were de-identified before conducting the analysis. The inclusion criteria included a confirmed diagnosis of small saccular aneurysm with a maximum diameter of 7 mm, availability of three-dimensional digital subtraction angiography (3D-DSA) data, and accessibility of clinical and radiological data. Patients with diagnoses of traumatic, infectious fusiform or dissecting aneurysms, malignant brain tumors, vascular malformations, arteriovenous fistulas, and moyamoya disease were excluded. The absence of clinical data or high-quality radiological data was also considered an exclusion criterion.

The criteria for unstable aneurysm were as follows: (a) ruptured aneurysm within 1 month and (b) growing aneurysm in a sequential imaging follow-up. Other unruptured aneurysms were categorized as stable. Thus, 709 IAs in 658 patients, including 293 unstable and 416 stable IAs, were included in this study.

### Clinical and Morphological Characteristics

The following clinical characteristics were collected from the medical records of each patient: age, sex, smoking, drinking, hypertension, hyperlipemia, diabetes mellitus, coronary heart disease, and family history of IA. The multiplicity and location of the IA were also recorded. The location of each IA was categorized as (a) internal carotid artery (ICA), (b) middle cerebral artery (MCA), (c) anterior cerebral artery (ACA), (d) anterior communicating artery (AComA), (e) posterior communicating artery (PComA), or (f) posterior circulation (PC) IAs.

To acquire precise, objective, and consistent measurements, the IA morphological features were extracted and measured from the reconstructed 3D-DSA. A three-dimensional model of each IA was reconstructed from the DSA into a standard tessellation language (STL) format and refined, as described in detail previously (14). Then, the morphological features were calculated using GEOMAGIC 12.0 software (Geomagic, Morrisville, North Carolina, USA) and Matlab (The Math Works, Inc., Natick, Massachusetts, USA) by two interventionalists who were blinded to the patient information and stability status. Discordance between the two evaluators was resolved by a third evaluator who has more than 10 years of experience in neuroradiology. Eighteen morphological features comprehensively describe the IAs and parent vessel geometries. These features included the maximum height (the maximum distance of the dome from the neck center), perpendicular height (the maximum perpendicular distance of the dome from the neck plane), neck diameter, width (the maximum perpendicular distance of the dome from the maximum height), transverse diameter (the maximum perpendicular distance of the dome from the perpendicular height), maximal diameter (the largest distance within the aneurysm sac, which is used as the size), and volume. In addition, the aspect ratio (AR), size ratio (SR), undulation index (UI), non-sphericity index (NSI), volume-to-neck ratio (VNR), height-to-width ratio, and bottleneck factor were defined and calculated as described in previous studies (15–17). Two features related to the parent vessel—the flow angle (angle between the vector of the IAs size and vector of the centerline of the feeding parent vessel) and aneurysm angle (angle between the plane of the neck and maximum height)—were also calculated. Irregular-shaped IAs were defined based on the presence of small bleb(s), bi- or multi-lobular, or protruding bulge(s) from the IAs fundus. The relative location of the IA dome to the parent vessel was classified as either sidewall or bifurcation type. Detailed descriptions of all features are provided in the online Supplementary Material.

### Statistical Analysis

For continuous variables, the differences between the groups were tested using the Student *t*-test or Mann–Whitney *U*-test. For categorical variables, a chi-square test was used to evaluate the differences between groups. Patient records were randomly divided into derivation (including 70% of the data) and validation data cohorts (including the remaining 30% of the data), which were used to develop and validate the nomogram, respectively. The ratio of unstable IAs to stable IAs was consistent between the derivation and validation cohorts.

In the derivation cohort, univariate and multivariate logistics regression analyses were used to screen the potential predictive factors. Statistically significant risk factors (*p* < 0.05) in the univariate analysis were considered for multivariate logistic regression analysis; then, risk factors with *p*-values under 0.05 in the multivariate logistic regression were distinguished for the model development. Moreover, according to Occam's law of razor, the best model for achieving optimal results is model with fewer variables. Accordingly, a receiver operating characteristic (ROC) curve and corresponding area under the curve (AUC) of each independent risk factor were calculated and compared to further simplify the model. As a result, a candidate nomogram model was formulated based on the most significant risk factors.

The predictive performance of the nomogram model to stratify IA instability was analyzed based on the ROC curve and corresponding AUC value. The fitness of the model was assessed by the Hosmer and Lemeshow test (*p* > 0.05 was considered to indicate a good fit) (18). Then, the nomogram was validated both internally in the derivation cohort and externally in the validation cohort. First, a calibration method with bootstrapping was utilized internally to illustrate the association between the actual IA status and the predicted instability probability. A calibration plot was created to show the apparent, bias-corrected, and ideal curves with bootstrapping samples. The external evaluation was carried out in the separate validation cohort based on the ROC curve and AUC and using the calibration plot. ROC curves were compared by the asymptotically exact method described by Delong et al. (19).

To adapt the model for clinical use, the total instability score of each IA was calculated based on the nomogram, and a ROC curve analysis was used to calculate the optimal cutoff values at which the Youden index (i.e., sensitivity + specificity −1) was maximized. The accuracy of the optimal cutoff value was assessed in terms of the sensitivity, specificity, and positive and negative predictive values.

Statistical analyses and figure plotting were carried out using R (version 3.6.1, R Foundation for Statistical Computing, Vienna, Austria). All tests were two-sided, and *p* ≤ 0.05 was considered statistically significant.

## Results

### Study Population

During the study period, 960 consecutive patients had confirmed diagnoses of small saccular aneurysms at our hospital. Of these, 709 IAs in 658 patients who met the inclusion criteria were enrolled. Of the IAs, 293 IAs were unstable, including 269 that ruptured, and 24 that grew in imaging follow-up (median: 12.3 months; range: 3–27 months). Five hundred nine IAs (including 213 unstable IAs) were randomly assigned to the derivation cohort, and the remaining 200 IAs (including 80 unstable IAs) were assigned to the validation cohort. Unstable IAs comprised 41.0% of the derivation cohort and 40.0% of the validation cohort. The clinical and morphological risk factors of the patients in derivation and validation cohorts are shown in Supplementary Table 1. The baseline data were similar between the two cohorts.

### Comparison of Clinical and Morphological Risk Factors of Stable and Unstable Aneurysms

The patient-related clinical risk factors of the derivation cohort and the whole population are shown in Supplementary Tables 1, 2, respectively. Hypertension, smoking, alcohol drinking, and multiplicity were common among those with unstable IAs (*p* < 0.05). Small IA instability was significantly associated with the IA location: the portions of unstable IAs located in the AComA and PC was higher than those of stable IAs in the same locations (31.5 vs. 8.8%, in the AComA; 6.1 vs. 2.7%, in the PC, respectively). In contrast, the stable IAs were more prevalent in the ICA than unstable ones (62.5 vs. 32.9% in the ICA). Further, 72.8% (155 of 242) of bifurcation IAs were unstable, representing higher instability in this subset than in the sidewall IAs subset.

The aneurysm-specific morphological risk factors of the derivation cohort and the whole population are shown in Supplementary Tables 1, 2, respectively. In the derivation cohort, the mean size of the unstable IAs was 5.06 ± 1.11 mm, which was not significantly different from that of the stable IAs (5.00 ± 1.16; *p* = 0.535). Other size indices (including the maximum height, width, and transverse diameter) and shape indices (such as the UI, NSI, VNR, and bottleneck factor) were significantly higher in the unstable IAs than in the stable IAs (*p* < 0.05). Moreover, a large proportion of unstable IAs had irregular shape compared with stable IAs (23.5 vs. 8.1%, *p* < 0.05). Compared with stable IAs, unstable IAs were more likely to be located at parent vessels with larger flow angles and smaller vessel diameters, which is consistent with the observed differences between rates of sidewall vs. bifurcation type and IAs location between the stable and unstable groups.

### Feature Selection and Nomogram Construction Based on the Derivation Cohort

Based on the univariate logistic regression analysis, 19 risk factors were determined to be statistically associated with IA instability (Table 1), while in the multivariate logistic analysis in which only variables with statistical significance were included based on the results of univariate analysis, we found that only location, irregular, sidewall/bifurcation type, multiplicity, flow angle, and height-to-width ratio were directly and independently linked to the IA instability (Table 1).

**Table 1:** Univariate and multivariate analysis of the derivation cohorts.

**Variables**	**Univariate**		**Multivariate**	
	**OR (95% CI)**	***p*-value**	**OR (95 % CI)**	***p*-value**
Age, years	0.990 (0.974, 1.007)	0.253	–	–
Gender (female), *n* (%)	0.787 (0.546, 1.135)	0.200	–	–
Hypertension, *n* (%)	1.933 (1.352, 2.762)	0.001	1.484 (0.944, 2.333)	0.087
Hyperlipidemia, *n* (%)	1.508 (0.817, 2.783)	0.189	–	–
Coronary heart disease, *n* (%)	1.625 (0.793, 3.331)	0.185	–	–
Diabetes mellitus, *n* (%)	0.947 (0.545, 1.645)	0.847	–	–
Smoker, *n* (%)	1.801 (1.194, 2.718)	0.005	1.248 (0.655, 2.377)	0.500
Drinker, *n* (%)	1.615 (1.072, 2.432)	0.022	1.174 (0.616, 2.238)	0.625
Family history, *n* (%)	1.961 (1.036, 3.714)	0.039	1.488 (0.655, 3.378)	0.343
Multiplicity, *n* (%)	0.449 (0.307, 0.658)	0.001	0.526 (0.335, 0.826)	0.005
Location, *n* (%)				0.025
ICA	Ref	Ref	Ref	Ref
MCA	2.379 (1.321, 4.283)	0.004	0.863 (0.418, 1.785)	0.692
ACA	2.265 (0.999, 5.136)	0.050	1.519 (0.599, 3.854)	0.379
AComA	6.810 (4.009, 11.569)	<0.001	1.604 (0.836, 3.079)	0.155
Posterior circulation	4.295 (1.707, 10.805)	0.002	4.421 (1.543, 12.668)	0.006
PComA	1.922 (1.062, 3.479)	0.031	1.949 (0.985, 3.856)	0.055
Sidewall/bifurcation, *n* (%)				0.001
Sidewall	Ref	Ref	Ref	Ref
Bifurcation	6.420 (4.339, 9.500)	<0.001	5.055 (3.021, 8.458)	0.001
Size	1.051 (0.899, 1.227)	0.534	–	–
Maximum height	1.215 (1.021, 1.445)	0.028	0.623 (0.192, 2.018)	0.430
Perpendicular height	1.113 (0.939, 1.321)	0.218	–	–
Neck diameter	0.737 (0.623, 0.873)	<0.001	1.072 (0.649, 1.769)	0.786
Width	0.785 (0.653, 0.944)	0.010	0.976 (0.296, 3.217)	0.969
Transverse diameter	0.818 (0.687, 0.974)	0.024	1.124 (0.581, 2.176)	0.728
Volume	0.990 (0.980, 1.001)	0.068	–	–
Aneurysm angle	0.991 (0.981, 1.000)	0.050	–	–
Flow angle	1.020 (1.013, 1.026)	<0.001	1.009 (1.001, 1.017)	0.023
AR	2.607 (1.498, 4.539)	0.001	1.233 (0.292, 5.214)	0.776
SR	2.559 (1.847, 3.545)	<0.001	0.754 (0.331, 1.717)	0.502
UI	5.291 (1.844, 9.42)	0.015	7.957 (2.687, 13.336)	0.231
NSI	2.024 (0.445, 9.201)	0.362	–	–
VNR	1.107 (0.965, 1.269)	0.146	–	–
Height to width ratio	9.361(4.397, 15.413)	<0.001	6.392 (2.433, 16.793)	0.001
Bottleneck factor	2.788 (1.466, 5.301)	0.002	2.102 (0.811, 5.452)	0.127
Vessel diameter	0.357 (0.274, 0.465)	<0.001	0.652 (0.355, 1.197)	0.167
Irregularity	3.476 (2.059, 5.871)	<0.001	2.707 (1.484, 4.938)	0.001

*ICA, internal carotid artery; MCA, middle cerebral artery; ACA, anterior cerebral artery; AComA, anterior communicating artery; PComA, posterior communicating artery; AR, aspect ratio; SR, size ratio; UI, undulation index; NSI, nonspherical index; VNR, volume-to-neck ratio*.

**Figure 1:**
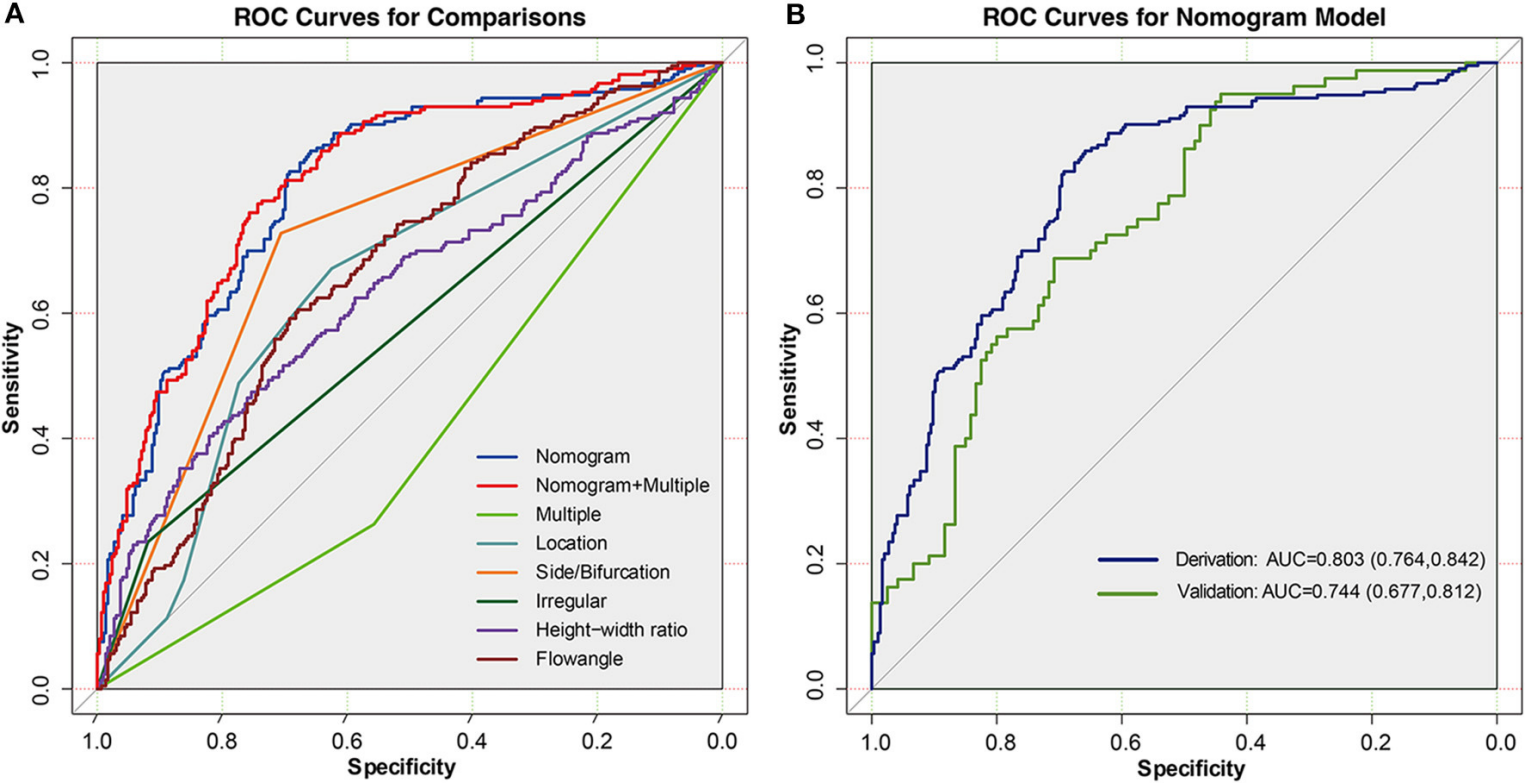
**(A)** ROC curves of the nomogram, nomogram combined with multiple, multiple, location, side/bifurcation type, irregular, height-width ratio, and flow angle in derivation cohort. **(B)** ROC curves in the derivation and validation cohorts for the nomogram. In the derivation cohort, the AUC of the nomogram model was 0.803 (95% CI = 0.764–0.842), and in the validation cohort the AUC was 0.744 (95% CI = 0.677–0.812).

**Figure 2:**
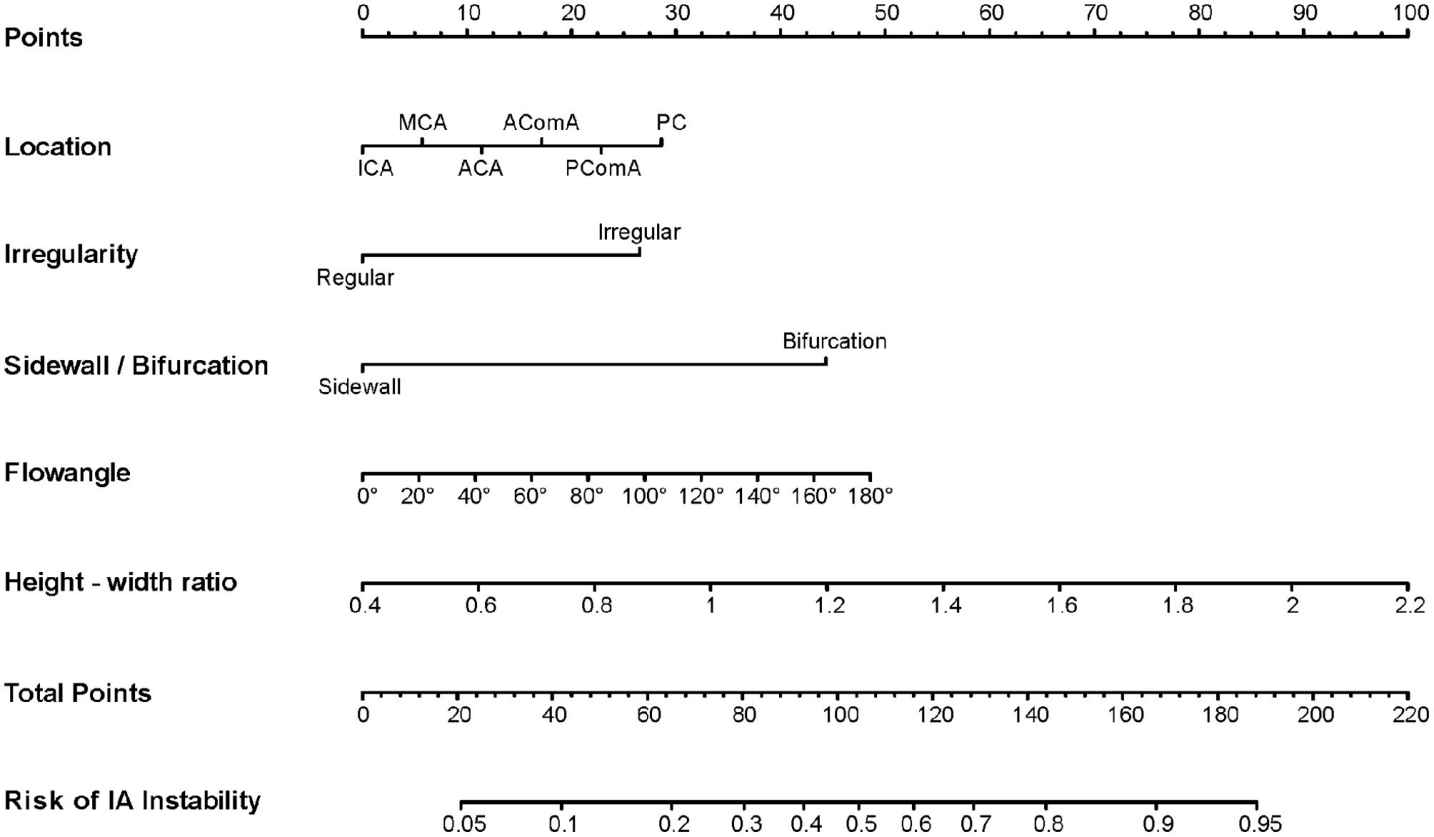
A nomogram to estimate the risk of small aneurysm instability based on preoperative clinical and morphological risk factors. The nomogram was developed in the derivation cohort based on five independent risk factors: location, irregularity, sidewall/bifurcation type, flow angle, and height-width ratio. To use the nomogram, find the position of each variable on the corresponding axis, draw a line to the points axis for the number of points, add the points from all of the variables, and draw a line from the total points axis to determine the risk of aneurysm instability at the lower line of the nomogram.

**Figure 3:**
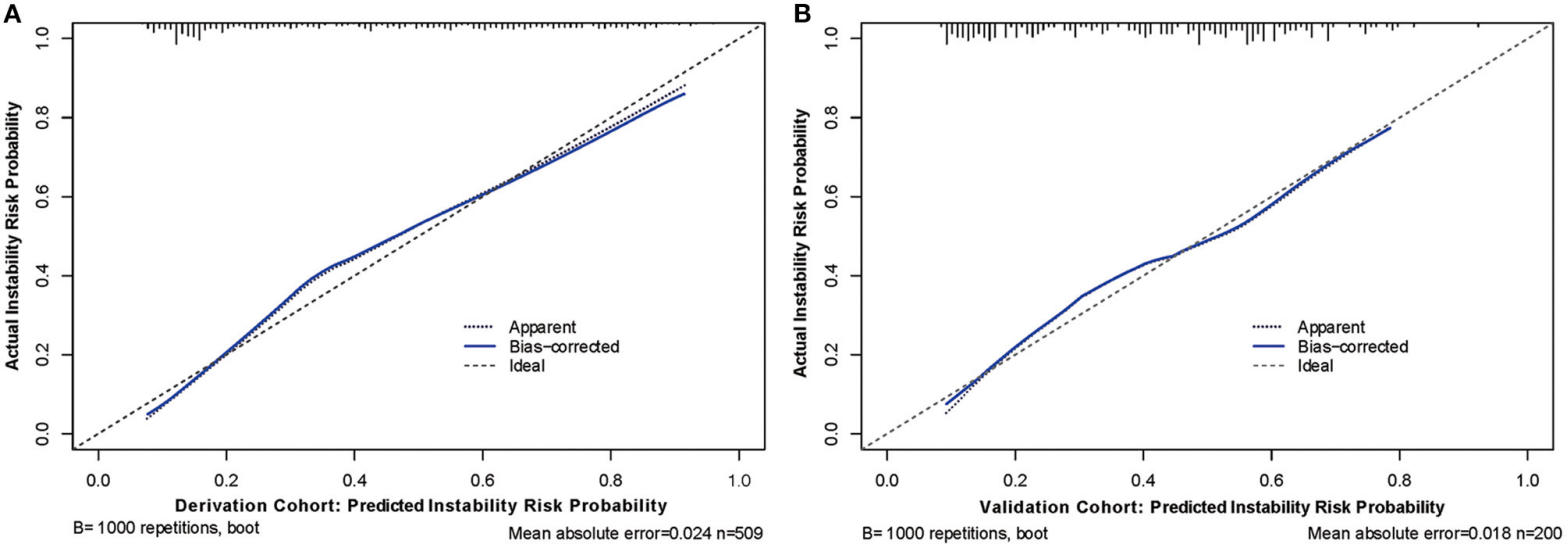
Calibration curves of the nomogram model with derivation **(A)** and validation **(B)** cohorts, respectively. Calibration curves depict the calibration of predictive model in terms of the agreement between the predicted risk of aneurysm instability and observed actual status of aneurysm stability. The *y* axis represents the actual status of aneurysm stability. The *x* axis represents the predicted probability of instability. The diagonal dotted line represents a perfect prediction by an ideal model. The blue solid line represents the performance of the nomogram, of which a closer fit to the diagonal line represents a better prediction.

Next, to formulate an optimal nomogram model, the individual and combined performances of these six factors were then comprehensively evaluated using ROC analysis. Figure 1A shows that the individual AUCs of the sidewall/bifurcation type, flow angle, height-to-width ratio, location, irregularity, and presence of multiple IAs were 0.717, 0.665, 0.634, 0.628, 0.577, and 0.410, respectively. Then, ROC curves with two combinations of factors were compared: the combination 1 (multiple, location, irregular, sidewall/bifurcation, flow angle, height-to-width ratio) and combination 2 (location, irregular, sidewall/bifurcation, flow angle, height-to-width ratio) performed similarly (AUC = 0.811 vs. 0.803, *p* = 0.11). To simplify the model, multiple was excluded from the model because of its relatively small AUC value (AUC = 0.410), which is significantly lower than other predictive factors' AUC values (ranging from 0.577 to 0.717). Hence, a nomogram for predicting the instability of small IAs was preliminarily constructed with these five risk factors: location, irregularity, sidewall/bifurcation, flow angle, and height-to-width ratio (Figures 1, 2). The AUC value of the nomogram was 0.803 (95% CI, 0.764–0.842), which indicated that the model had good discriminatory ability. A Hosmer and Lemeshow test yielded a *p*-value of 0.092, indicating the model was also well-fitted. Furthermore, we did the calibration plot of the nomogram internally with bootstrap sampling with 1,000 iterations. The results in Figure 3A reveal good agreement between the actual instability status and predicted IA instability risk estimated by the nomogram.

### Nomogram Validation in the Validation Cohort

To further verify the efficacy and generalizability of the developed nomogram to predict IAs instability, we conducted comprehensive validations in a separate validation cohort. The result in Figure 1B indicates that the AUC value of the nomogram was 0.744 (95% CI, 0.677–0.812) in the validation cohort. Further, the calibration curve in Figure 3B indicates that there was a good agreement between the actual instability status and the IA instability risk estimated using the nomogram in the validation cohort. Finally, there was no statistically significant difference between the AUCs of the derivation and validation cohorts (*p* = 0.144). These results demonstrate that the good performance of the nomogram model is generalizable beyond the derivation cohort.

### Clinical Use of Nomogram Scores to Predict Instability

Based on the derivation cohort, the optimal cutoff value of the nomogram scores was determined to be 90. Thus, the total scores calculated using the nomogram were used to categorized all IAs into two IA instability risk groups: the low-risk group (<90) and the high-risk group (>90). In the differentiation of unstable small IAs from unruptured small IAs, the sensitivity, specificity, positive predictive value, and negative predictive value were 82.6, 69.3, 65.9, and 84.7%, respectively, in the derivation cohort and 78.8, 52.5, 52.5, and 78.8%, respectively, in the validation cohort (Table 2).

**Table 2:** Accuracy of the prediction score of the nomogram for estimating the risk of IAs instability.

**Variable**	**Derivation cohort**	**Validation cohort**
Area under the curve	0.803 (0.764, 0.842)	0.744 (0.677, 0.812)
Cutoff score	90	90
Sensitivity	82.6%	78.8%
Specificity	69.3%	52.5%
Positive predictive value	65.9%	52.5%
Negative predictive value	84.75%	78.8%

## Discussion

In this study, we developed and validated a predictive nomogram based on five risk factors: location, irregularity, sidewall/bifurcation type, flow angle, and height-to-width ratio. The nomogram model provides accurate instability stratification, as evidenced by its discriminative ability and the calibration plots, and can be used to aid clinicians, patients, and families in decision-making in cases of small, unruptured IAs.

In the present study, although the IAs in the unstable cohort were larger than those in the stable cohort, this factor was not retained in the nomogram as the other important risk factors were. A possible reason is that the instability of small IAs may be closely related to their shape and locations relative to the parent vessels rather than size. As the nomogram indicated, the irregular shape and height-to-width ratio are the two most important risk factors of the 18 morphological characteristics evaluated here. Those two factors are commonly used indicators of IAs shape complexity and have been consistently proven to be consistently associated with IAs instability in previous studies (17, 20). Ryu et al. reported that ruptured IAs typically exhibited higher height-width ratio (near 1.0) (15). From the perspective of the hemodynamic analysis, regular IAs and those with low height-to-width ratio exhibit simple flow dynamics, involving a constant flow jet direction with a single associated vortex; in contrast, irregular IAs and those with higher height-to-width ratio tend to have a complex flow dynamics, involving a varied inflow jet with multiple vortices. This difference may reflect a possible mechanism underlying instability in small IAs (21). The flow angle and sidewall/bifurcation type are two other independent risk factors that were found to be associated with relationship between the IAs dome and the parent vessels, for which their spatial relationship has been shown to be an important determinant of flow patterns inside the IA dome (22, 23). Computational fluid dynamic analysis showed that larger flow angel and bifurcation location are always associated with stronger blood flow and elevated hemodynamic stress, both of which constantly damage the arterial wall and accelerate the deterioration and remodeling process of the arterial wall and ultimately cause the IA to grow or rupture (24, 25).

Considering the IA location, we found that IAs located at the ICA have decreased instability propensity than those in other locations, while IAs located in the PC are more likely to be unstable. These findings are consistent with previous studies (26). Varble et al. suggested that IAs in locations other than the ICA are subjected to lower wall shear stress compared with those at the ICA, which may result in higher rupture risk of those IAs (27).

The use of nomogram for IAs instability stratification is a new concept. Previous studies have attempted to build prediction models based on clinical and morphological risk factors for IAs stratification. Scoring systems, such as the PHASES (population, hypertension, age, size, early hemorrhage history, and sites) score and UIATS (unruptured intracranial aneurysm treatment score) score, have been reported (26, 28). Although these scoring systems are based on clinical and morphological factors and could be easily used in typical clinical settings, it is necessary to improve their performances. Moreover, those scoring systems were not designed specifically for small IAs. Given the distinctive pathophysiological presentations between large and small IAs (29, 30). It is important to develop a sized-specific model for instability stratification separately. Several studies have investigated the use of machine learning (ML) algorithms to assess the instability of small IAs. Liu et al. developed an ML model based on clinical risk factors and PyRadiomics-derived morphological features, and the AUC value of this ML model reached 0.853 (31). Kim et al. constructed a system to predict IA instability from 3D-DSA images based on a convolutional neural network; the system exhibited a sensitivity of 78.76%, a specificity of 72.15%, and an AUC value of 0.755 (32). However, further clinical validation is required before these ML algorithms can be implemented. Furthermore, the use of ML algorithms requires specific computer software, and it cannot be run on handheld devices, thus limiting its widespread use. Among the currently available prediction tools, nomograms are highly accurate, offer good discriminatory ability, and are easy to use. In the present study, the developed nomogram that incorporated five easily accessible and comprehensive factors performed well, as evidenced by AUC values of 0.803 and 0.744 in the derivation and validation cohorts, respectively, and calibration curves demonstrating good agreements between the predicted IA instability risk and actual IA status.

To demonstrate the potential for this nomogram to be used clinically, the sensitivity, specificity, positive predictive value, and negative predictive value in estimating the instability of small IAs using an optimized cutoff value of 90 are summarized in Table 2. IAs with scores of 90 or higher are considered to have high risk of instability. In addition, using 90 as the cutoff value, the sensitivity (82.6%) prioritizes the corresponding specificity (69.3%), demonstrating another advantage of the nomogram model, because high sensitivity is especially important for a stratification model intended to identify IAs, as they have high morbidity and mortality of aneurysmal SAH. However, it is important to note that the IA management decision-making process is multifactorial and complicated. Thus, in clinical practice, the ideal choice of cutoff value for treatment decision-making depends on the preferences and judgement of the surgeons and the patient's specific conditions. This nomogram serves as an easy-to-use supportive tool that can facilitate individualized stratification of IA instability and assist the decision-making process for small IAs management.

## Limitations

Our study had several limitations. First, this study involved only a Chinese single-center population. Some studies indicate Japanese and Finnish populations have a higher rupture risk (26). Consequently, the model's performance could be different in test data from these populations. Future work therefore will aim at evaluating the nomogram performance with such data. Second, this study was retrospective, which is associated with an inherent risk of bias; a prospective study is required to further confirm the reliability of the nomogram. Third, the event of rupture itself may directly affect the IA morphology, which may also generate a possible bias in our analysis. Finally, the model was based only on clinical and morphological risk factors, but other promising risk factors, such as the vessel wall parameters observed by other imaging studies, may be incorporated in the future to further improve the model performance.

## Conclusions

Our findings suggest that the constructed nomogram based on clinical and morphological risk factors can be used for instability stratification of small IAs. This model can be conveniently used to facilitate the individualized decision-making process to manage IAs.

## Data Availability Statement

The raw data supporting the conclusions of this article will be made available by the authors, without undue reservation.

## Ethics Statement

The studies involving human participants were reviewed and approved by Institutional Review Board of Beijing Tiantan Hospital. The requirement for written informed consent was waived due to the retrospective nature of the study.

## Author Contributions

JL and XY: conceptualization. WL and MZ: data collection. YinZ and YisZ: data curation. ZT: investigation. WZ: methodology and writing. All authors contributed to the article and approved the submitted version.

## Conflict of Interest

The authors declare that the research was conducted in the absence of any commercial or financial relationships that could be construed as a potential conflict of interest.

## Abbreviations

IA: intracranial aneurysm
SAH: subarachnoid hemorrhage
ROC: receiver operating curve
AUC: area under the curve
CI: confidence interval
ICA: internal carotid artery
MCA: middle cerebral artery
ACA: anterior cerebral artery
AComA: anterior communicating artery
PComA: posterior communicating artery
PC: posterior circulation.
