# Supplementary material for: Nomogram for Stability Stratification of Small Intracranial Aneurysm Based on Clinical and Morphological Risk Factors
Source: Front Neurol. 2021 Jan 15;11:598740. doi: 10.3389/fneur.2020.598740 (PMC7845661; doi:10.3389/fneur.2020.598740)
Supplement: Supplementary file 1 [file Data_Sheet_1.docx]

**Supplemental Material**

**Supplemental Method**

**Definitions of Morphological features**

Morphological features were extracted and measured from the reconstructed three-Dimensional (3D) DSA, morphological features involved in this study are listed below:

Max diameter: defined as the largest distance within aneurysm sac from cross-sectional projection, used as the size.

Maximum height: defined as the maximum distance of the dome from the neck center.

Perpendicular Height: defined as the maximum perpendicular distance of the dome from the neck plane.

Neck diameter: defined as the maximum diameter of the aneurysm neck plane.

Aneurysm Width: the maximum distance of dome perpendicular to maximum height.

Transverse Diameter: defined as the maximum distance of dome perpendicular to perpendicular height:

Aneurysm Volume: the volume of aneurysm.

Aneurysm angle: defined as angles between plane of neck and maximum height.^[1]^

Flow angle: defined as angles between the vector of maximum height and vector of centerline of feeding parent vessel.^[2]^

Aspect ratio (AR): defined as the ratio of maximum height to neck diameter.^[3]^

Size ratio (SR): defined as maximum height / vessel diameter.^[1]^

Undulation index (UI): UI=1 - (V/Vch), where V is the volume of the aneurysm above the neck plane and Vch is the volume of the convex hull. The convex hull of the IA is the smallest volume that fully encloses the IA volume and that is convex at all points.^[4]^

Nonsphericity index (NSI): defined as NSI =1 - (18π)1/3 · V2/3/ S . V is the volume of IA, S is the surface area of IA. NSI will vary from 0 to 1. It is equal to 0 for a hemisphere and increases with increasing deviation from the spherical shape, be it due to undulation or ellipticity.^[4]^

Volume to neck ratio: defined as ratio of volume to neck area.^[5]^

Height-width ratio: defined as the ratio of maximum height to width ratio.

Bottleneck factor: defined as the ratio of transverse diameter to neck diameter.^[6]^

Vessel diameter: defined as the diameter of parent vessel.

Irregularity: defined as the presence of small bleb(s), bi-or multi-lobular, protruding bulge(s) from the IA fundus.

**
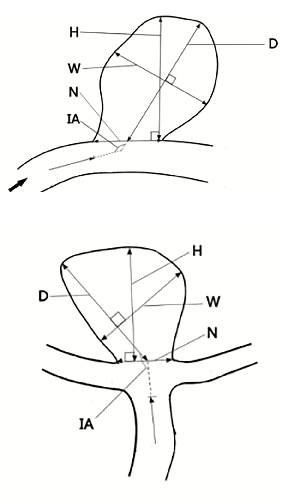
**

**Fig I .** Measurement of aneurysm morphological features: *D,* maximum height; H, perpendicular height; W, aneurysm width; N, aneurysm neck diameter; IA, inflow angle.

**Supplemental Table**

Table I. Clinical and Aneurysm Morphological Characteristics of the Derivation and Validation Cohorts

| Characteristics | Derivation | Validation | *P* value |
| --- | --- | --- | --- |
| Unstable, n (%) | 213 (41.8) | 80 (40.0) | 0.672 |
| Age,y | 54.47 ± 10.69 | 54.09 ± 10.28 | 0.658 |
| Gender (female), n (%) | 327 (64.2) | 138 (69) | 0.254 |
| Hypertension, n (%) | 248 (48.7) | 108 (54) | 0.212 |
| Hyperlipidemia, n (%) | 45 (8.8) | 16 (8) | 0.768 |
| Diabetes mellitus, n (%) | 59 (11.6) | 26 (13) | 0.609 |
| Coronary heart disease, n (%) | 32 (6.3) | 21 (10.5) | 0.061 |
| Smoker, n (%) | 121 (23.8) | 43 (21.5) | 0.554 |
| Drinker, n (%) | 122 (24.0) | 48 (24) | 0.532 |
| Family history, n (%) | 42 (8.3) | 18 (9) | 0.765 |
| Multiple, n (%) | 187 (36.7) | 78 (39) | 0.605 |
| Location, n (%) |  |  | 0.081 |
| ICA | 255 (50.1) | 82 (41) |  |
| MCA | 57 (11.2) | 26 (13) |  |
| ACA | 26 (5.1) | 7 (3.5) |  |
| AComA | 93 (18.3) | 49 (24.5) |  |
| Posterior Circulation | 21 (4.1) | 20 (10) |  |
| PComA | 57 (11.2) | 16 (8) |  |
| Sidewall/Bifurcation, n (%) |  |  | 0.054 |
| Sidewall | 267 (52.5) | 88 (44) |  |
| Bifurcation | 242 (47.5) | 112 (56) |  |
| Irregularity n (%) | 74 (14.5) | 20 (10.0) | 0.057 |
| Size, mm | 5.03 ± 1.14 | 5.08 ± 1.17 | 0.563 |
| Maximum height, mm | 3.61 ± 1.03 | 3.64 ± 1.00 | 0.647 |
| Perpendicular height, mm | 3.15 ± 1.04 | 3.21 ± 1.01 | 0.458 |
| Neck diameter, mm | 4.35 ± 1.09 | 4.31 ± 1.10 | 0.693 |
| Aneurysm Width, mm | 4.03 ± 0.98 | 4.01 ± 1.01 | 0.855 |
| Transverse Diameter, mm | 4.19 ± 1.02 | 4.16 ± 1.09 | 0.660 |
| Aneurysm Volume, mm^3^ | 26.35 ± 17.37 | 27.12 ± 17.97 | 0.601 |
| Aneurysm angle | 54.20 ± 18.98 | 54.70 ± 18.74 | 0.751 |
| Flow angle | 102.26 ± 31.44 | 103.88 ± 31.37 | 0.538 |
| AR | 0.77 ± 0.34 | 0.79 ± 0.34 | 0.470 |
| SR | 1.34 ± 0.61 | 1.44 ± 0.66 | 0.066 |
| UI | 0.17 ± 0.07 | 0.16 ± 0.06 | 0.528 |
| NSI | 0.29 ± 0.12 | 0.29 ± 0.11 | 0.474 |
| Volume to neck ratio | 1.81 ± 1.30 | 1.85 ± 1.24 | 0.740 |
| Height-width ratio | 0.91 ± 0.22 | 0.93 ± 0.24 | 0.263 |
| Bottleneck factor | 0.85 ± 0.28 | 0.85 ± 0.31 | 0.929 |
| Vessel diameter, mm | 2.96 ± 0.79 | 2.80 ± 0.83 | 0.053 |

ICA: internal carotid artery; MCA: middle cerebral artery; ACA: anterior cerebral artery; AComA: anterior communicating artery; PComA: posterior communicating artery; AR: aspect ratio; SR: size ratio; UI: undulation index; NSI: nonspherical index.

Table II. Demographic and Clinical Characteristics Between Stable and Unstable Groups in Derivation Cohort

|  | Stable  (n= 296) | Unstable  (n= 213) | *P* value |
| --- | --- | --- | --- |
| Age, y | 54.93 ± 10.12 | 53.83 ± 11.40 | 0.263 |
| Gender (female), n (%) | 197 (66.6) | 130 (61.0) | 0.223 |
| Hypertension, n (%) | 124 (41.9) | 124 (58.2) | < 0.001 |
| Hyperlipidemia, n (%) | 22 (7.4) | 23 (10.8) | 0.207 |
| Coronary heart disease, n (%) | 15 (5.1) | 17 (8.0) | 0.198 |
| Diabetes mellitus, n (%) | 35 (11.8) | 24 (11.3) | 0.889 |
| Smoker, n (%) | 57 (19.3) | 64 (30.0) | 0.006 |
| Drinker, n (%) | 60 (20.3) | 62 (29.1) | 0.027 |
| Family history, n (%) | 18 (6.1) | 24 (11.3) | 0.053 |
| Multiplicity, n (%) | 131 (44.3) | 56 (26.3) | < 0.001 |
| Location, n (%) |  |  | < 0.001 |
| ICA | 185 (62.5) | 70 (32.9) |  |
| MCA | 30 (10.1) | 27(12.7) |  |
| ACA | 14 (4.7) | 12 (5.6) |  |
| AComA | 26 (8.8) | 67 (31.5) |  |
| Posterior Circulation | 8 (2.7) | 13 (6.1) |  |
| PComA | 33 (11.1) | 24 (11.3) |  |
| Sidewall/Bifurcation, n (%) |  |  |  |
| Sidewall | 209 (70.6) | 58 (27.2) | < 0.001 |
| Bifurcation | 87 (29.4) | 155 (72.8) |  |

Table III. Morphological Characteristics Between Stable and Unstable Groups in Derivation Cohort

|  | Stable  (n= 296) | Unstable  (n= 213) | *P* value |
| --- | --- | --- | --- |
| Size | 5.00 ± 1.16 | 5.06 ± 1.11 | 0.535 |
| Maximum height | 3.52 ± 1.00 | 3.72 ± 1.05 | 0.027 |
| Perpendicular height | 3.10 ± 1.00 | 3.21 ± 1.08 | 0.218 |
| Neck diameter | 4.50 ± 1.10 | 4.14 ± 1.06 | 0.001 |
| Width | 4.12 ± 0.99 | 3.89 ± 0.95 | 0.010 |
| Transverse diameter | 4.28 ± 1.02 | 4.07 ± 1.01 | 0.024 |
| Volume | 27.55 ± 18.39 | 24.70 ± 15.74 | 0.067 |
| Aneurysm angle | 55.62 ± 18.39 | 52.24 ± 19.40 | 0.051 |
| Flow angle | 94.81 ± 31.99 | 112.61 ± 27.55 | <0.001 |
| AR | 0.73 ± 0.31 | 0.83 ± 0.37 | <0.001 |
| SR | 1.20 ± 0.53 | 1.53 ± 0.70 | <0.001 |
| UI | 0.16 ± 0.07 | 0.18 ± 0.07 | 0.014 |
| NSI | 0.28 ± 0.12 | 0.30 ± 0.12 | 0.362 |
| VNR | 1.74 ± 1.30 | 1.91 ± 1.28 | 0.142 |
| Height to width ratio | 0.89 ± 0.19 | 0.97 ± 0.25 | <0.001 |
| Bottleneck factor | 0.81 ± 0.28 | 0.89 ± 0.32 | 0.001 |
| Vessel diameter | 3.20 ± 0.76 | 2.63 ± 0.76 | <0.001 |
| Iregularity | 24 (8.1) | 50 (23.5) | <0.001 |

Table IV. Demographic and Clinical characteristics Between Stable and Unstable Groups in Validation Cohort

|  | Stable  (n= 120) | Unstable  (n= 80) | *P* value |
| --- | --- | --- | --- |
| Age, y | 54.18 ± 9.20 | 53.95 ± 11.77 | 0.880 |
| Gender (female), n (%) | 80 (66.7) | 58 (72.5) | 0.437 |
| Hypertension, n (%) | 61 (50.8) | 47 (58.8) | 0.312 |
| Hyperlipidemia, n (%) | 11 (9.2) | 5 (6.3) | 0.597 |
| Coronary heart disease, n (%) | 10 (8.3) | 11 (13.8) | 0.245 |
| Diabetes mellitus, n (%) | 18 (15.0) | 8 (10.0) | 0.392 |
| Smoker, n (%) | 23 (19.2) | 20 (25.0) | 0.381 |
| Drinker, n (%) | 27 (22.5) | 21 (26.3) | 0.613 |
| Family history, n (%) | 8 (6.7) | 10 (12.5) | 0.208 |
| Multiplicity, n (%) | 53 (44.2) | 25 (31.3) | 0.077 |
| Location, n (%) |  |  | 0.001 |
| ICA | 61 (50.8) | 21 (26.3) |  |
| MCA | 16 (13.3) | 10 (12.5) |  |
| ACA | 6 (5.0) | 1 (1.3) |  |
| AComA | 17 (14.2) | 32 (40.0) |  |
| Posterior Circulation | 12 (10.0) | 8 (10.0) |  |
| PComA | 8 (6.7) | 8 (10.0) |  |
| Sidewall/Bifurcation, n (%) |  |  |  |
| Sidewall | 68 (56.7) | 20 (25.0) | < 0.001 |
| Bifurcation | 52 (43.3) | 60 (75.0) |  |
|  |  |  |  |

Table V. Morphological Characteristics Between Stable and Unstable Groups in Validation Cohort

|  | Stable  (n= 120) | Unstable  (n= 80) | *P* value |
| --- | --- | --- | --- |
| Size | 5.04 ± 1.16 | 5.14 ± 1.17 | 0.560 |
| Maximum height | 3.48 ± 0.88 | 3.89 ± 1.13 | 0.004 |
| Perpendicular height | 3.08 ± 0.89 | 3.40 ± 1.15 | 0.028 |
| Neck diameter | 4.51 ± 1.10 | 4.01 ± 1.02 | 0.001 |
| Width | 4.10 ± 1.07 | 3.88 ± 0.92 | 0.125 |
| Transverse diameter | 4.26 ± 1.51 | 4.00 ± 0.96 | 0.109 |
| Volume | 28.58 ± 20.25 | 24.93 ± 13.70 | 0.160 |
| Aneurysm angle | 55.41 ± 18.54 | 53.65 ± 19.11 | 0.520 |
| Flow angle | 98.20 ± 31.32 | 112.38 ± 29.64 | 0.002 |
| AR | 0.72 ± 0.29 | 0.89 ± 0.37 | <0.001 |
| SR | 1.27 ± 0.51 | 1.66 ± 0.78 | <0.001 |
| UI | 0.15 ± 0.06 | 0.18 ± 0.06 | 0.003 |
| NSI | 0.27 ± 0.12 | 0.30 ± 0.10 | 0.072 |
| VNR | 1.71 ± 1.13 | 2.06 ± 1.38 | 0.059 |
| Height to width ratio | 0.87 ± 0.20 | 1.02 ± 0.27 | <0.001 |
| Bottleneck factor | 0.82 ± 0.29 | 0.89 ± 0.32 | 0.098 |
| Vessel diameter | 3.03 ± 0.86 | 2.43 ± 0.63 | <0.001 |
| Iregularity | 7 (5.8) | 9 (11.3) | 0.190 |
|  |  |  |  |

**Supplemental Reference**

1. Dhar, S., M. Tremmel, J. Mocco, et al., *Morphology parameters for intracranial aneurysm rupture risk assessment.* Neurosurgery, 2008. **63**(2): p. 185-96; discussion 196-7.

2. Ho, A., N. Lin, N. Charoenvimolphan, et al., *Morphological parameters associated with ruptured posterior communicating aneurysms.* PloS one, 2014. **9**(4): p. e94837-e94837.

3. Ujiie, H., Y. Tamano, K. Sasaki, et al., *Is the aspect ratio a reliable index for predicting the rupture of a saccular aneurysm?* Neurosurgery, 2001. **48**(3): p. 495-502; discussion 502-3.

4. Raghavan, M.L., B. Ma, and R.E. Harbaugh, *Quantified aneurysm shape and rupture risk.* J Neurosurg, 2005. **102**(2): p. 355-62.

5. Ryu, C.-W., O.K. Kwon, J.S. Koh, et al., *Analysis of aneurysm rupture in relation to the geometric indices: aspect ratio, volume, and volume-to-neck ratio.* Neuroradiology, 2010. **53**(11): p. 883-889.

6. Hoh, B.L., C.L. Sistrom, C.S. Firment, et al., *Bottleneck factor and height-width ratio: association with ruptured aneurysms in patients with multiple cerebral aneurysms.* Neurosurgery, 2007. **61**(4): p. 716-22; discussion 722-3.
